# Electroacupuncture as Adjunctive Therapy for Organ Dysfunction Among Chinese ICU Patients with Sepsis: Study Protocol for a Randomized Clinical Trial

**DOI:** 10.1007/s11596-026-00225-5

**Published:** 2026-07-23

**Authors:** Qi Meng, Ling-ling Yu, Xiang-hong Jing, Man Li, Qian-rui Huang, Ting-ting Xu, Yi Bian, Shu-sheng Li

**Affiliations:** 1 Department of Critical Care Medicine, Tongji Hospital, Tongji Medical College, Huazhong University of Science and Technology, Wuhan, 430030 China; 2 Department of Emergency Medicine, Tongji Hospital, Tongji Medical College, Huazhong University of Science and Technology, Wuhan, 430030 China; 3 Institute of Integrated Traditional Chinese and Western Medicine, Tongji Hospital, Tongji Medical College, Huazhong University of Science and Technology, Wuhan, 430030 China; 4 Institute of Acupuncture and Moxibustion, China Academy of Chinese Medical Sciences, Beijing, 100007 China; 5 School of Basic Medicine, Tongji Medical College, Huazhong University of Science and Technology, Wuhan, 430030 China

**Keywords:** Sepsis, Electroacupuncture, Organ dysfunction, Inflammation, Randomized controlled trial

## Abstract

**Background:**

Sepsis is a dysregulated response of the host to infection that can lead to life-threatening organ dysfunction and remains a leading cause of mortality. Early recovery of impaired organ function is crucial for the outcomes of sepsis. Electroacupuncture (EA) is a promising adjunctive therapy for sepsis, but no study has focused on the efficacy of EA for organ dysfunction. This study aimed to investigate the efficacy and safety of EA in improving organ dysfunction among intensive care unit (ICU) patients with sepsis.

**Methods:**

In this single-center, randomized, sham-controlled clinical trial, an adaptive sample size of 154–500 patients diagnosed with sepsis will be included. Eligible patients will be randomly assigned to receive EA or sham EA treatment at a 1:1 ratio using block randomization. Patients in the two groups will receive 6 consecutive sessions of 30-min EA or sham EA treatment, with the initial acupuncture treatment being completed within 24 h after randomization. The primary outcome is the change in the total score of the Sepsis-Related Sequential Organ Failure Assessment (SOFA) on day 7 after randomization compared with baseline. Secondary outcomes include changes in the SOFA score and related serological outcomes, mortality, ICU-free days, hospital-free days and organ support-free days from baseline to day 28. Statistical analyses will be performed on a full analysis set (FAS) and a per-protocol set (PPS).

**Discussion:**

We expect the findings of this trial to provide evidence that the use of EA as an adjunctive therapy to ICU treatment can promote the recovery of organ function and other outcomes in sepsis patients.

**Trial Registration:**

ClinicalTrials.gov identifier: NCT06666946. Registered on 28 October 2024.

**Supplementary Information:**

The online version contains supplementary material available at 10.1007/s11596-026-00225-5.

## Introduction

Sepsis is a life-threatening condition characterized by a dysregulated response to infection and can lead to multiorgan dysfunction or septic shock [1]. It is a major cause of mortality, and the latest Global Burden of Disease Study reported 11.0 million sepsis-related deaths in 2017, representing 19.7% of all global deaths [2]. Moreover, sepsis imposes a significant burden on healthcare systems worldwide, with approximately half of sepsis patients receiving intensive care unit (ICU) care [3]. In China, sepsis is associated with an increasing burden of hospitalizations and affects one-fifth of patients admitted to ICUs [4, 5]. Therefore, the World Health Organization (WHO) considers sepsis a global public health priority [6].

The underlying mechanisms of sepsis involve excessive inflammation and immune suppression, resulting in multiorgan dysfunction or even septic shock [7, 8]. Although numerous interventions targeting the attenuation of inflammation or the removal of inflammatory mediators, such as glucocorticoids, vitamin C and some new targeted drugs, have shown promise in theory, most randomized controlled clinical trials (RCTs) have failed to demonstrate significant reductions in mortality or improvements in other clinical outcomes of sepsis [9–18]. Sepsis patients rarely exhibit single-organ dysfunction; instead, they typically progress to multiple-organ failure, in which the severity strongly correlates with the mortality of sepsis [19]. However, most sepsis-induced organ dysfunction is reversible [20]. Thus, the development of new therapies to prevent or mitigate organ dysfunction and prevent cytokine storms caused by sepsis is highly necessary.

Acupuncture has emerged as an adjunctive therapy for sepsis because of its potential efficacy in reducing inflammation or facilitating immune normalization [21–23]. Electroacupuncture (EA) is an adaptation of traditional acupuncture, and the core mechanism through which EA is used to treat various diseases is the delivery of controlled electrical pulses through fine needles inserted at specific acupuncture points. Notably, the use of acupuncture and EA is currently transitioning from empirical medicine to evidence-based medicine [24]. Recently, a study published in Neuron demonstrated that low-intensity EA can effectively inhibit excessive inflammatory responses and reduce the 7-day mortality rate by approximately 40% [25]. A subsequent study published in Nature further confirmed that this effect is mediated through the “PROKR2^ADV^ fibers-vagus nerve-adrenal NPY^+^ chromaffin cells” pathway [21]. These seminal findings indicate the promising role of EA as an adjunctive therapy for sepsis. In addition, several small-sample clinical trials have provided preliminary evidence supporting the efficacy of EA for sepsis [23]. However, limited evidence has supported the efficacy of EA for preventing organ dysfunction induced by sepsis. Therefore, this trial aimed to investigate whether EA, as an adjunctive treatment to standard-of-care therapy, can promote the recovery of organ function and other outcomes of sepsis patients in the ICU. The study protocol is designed in response to the core recommendations of the Rome Consensus on Good Clinical Trials for Traditional Medicine [26].

## Methods and Analysis

### Study Design

**Fig. 1:**
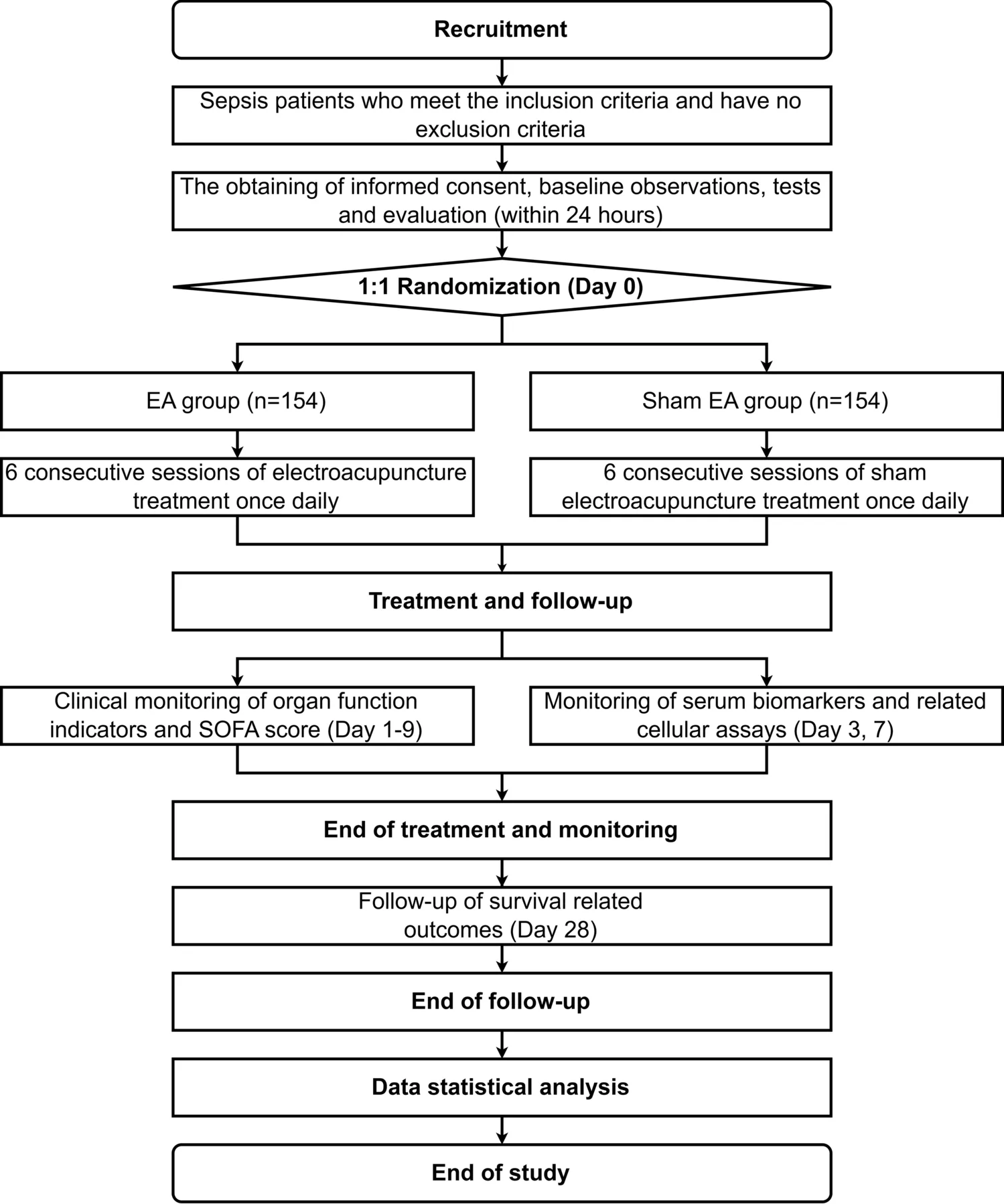
Study flow diagram

This is a single-center, randomized, participant- and outcome-assessor-blinded, sham-controlled, clinical study. The study will be conducted at the Tongji Hospital, affiliated with Tongji Medical College, Huazhong University of Science and Technology (HUST) in Wuhan, China. An adaptive sample size of 154–500 patients will be recruited from the ICU ward within 24 h of their ICU admission. The total trial period comprises 24 h of baseline assessment, 6 days of postrandomization treatment, and a 22-day follow-up period. Written informed consent will be obtained before randomization. ICU-attending physicians will provide detailed informed consent to patients or their legal guardians under ICU monitoring. Upon confirmation of eligibility, patients will be randomized into either the EA group or the sham-EA group. A complete treatment protocol requires the following: (1) the initial acupuncture treatment should be administered within 12 h post-randomization, and (2) six sessions of EA or sham EA should be conducted once daily for 30 min per session. The study protocol strictly adhered to the Standard Protocol Items: Recommendations for Interventional Trials (SPIRIT) reporting guideline [27]. The trial flowchart is presented in Fig. 1, with detailed procedures and schedules outlined in Table 1.

**Table 1:** Schedule of recruitment, interventions, and measures

	Time point (days)											
	Baseline		Treatment						Follow-up			
	–1	0	1	2	3	4	5	6	7	8	9	28
**Enrollment of sepsis patients**												
Screening	** × **											
Informed consent	** × **											
Demographic characteristics	** × **											
Previous disease history	** × **											
Physical examination	** × **											
**Randomization**		** × **										
**Intervention**												
EA group			** × **	** × **	** × **	** × **	** × **	** × **				
Sham EA group			** × **	** × **	** × **	** × **	** × **	** × **				
**Measures**												
SOFA score	** × **		** × **	** × **	** × **	** × **	** × **	** × **	** × **	** × **	** × **	
SOFA score of each system	** × **		** × **	** × **	** × **	** × **	** × **	** × **	** × **	** × **	** × **	
SOFA score components	** × **		** × **	** × **	** × **	** × **	** × **	** × **	** × **	** × **	** × **	
All-cause (ICU) mortality												** × **
ICU/hospital-free days												** × **
Ventilator-free time												** × **
Vasopressor-free days												** × **
CRRT-free days												** × **
Liquid equilibrium parameter	** × **		** × **	** × **	** × **	** × **	** × **	** × **				
Serum biomarkers and related cellular assays	** × **				** × **				** × **			
AEs			** × **	** × **	** × **	** × **	** × **	** × **				

EA, electroacupuncture; SOFA score, Sequential Organ Failure Assessment score; ICU, intensive care unit; CRRT, continuous renal replacement therapy; AEs, adverse events

### Eligibility Criteria

#### Inclusion Criteria

Eligibility patients have to meet the following inclusion criteria: 1) 18 years or older, no restriction on sex; 2) diagnosis of sepsis on the basis of Sepsis-3 criteria: an increase in the Sepsis-Related Sequential Organ Failure Assessment (SOFA) score by more than 2 points from baseline in the context of confirmed or suspected infection [1]; and 3) the patient or their legal guardian having the ability to understand the informed consent form and willingness to sign it.

#### Exclusion and Shedding Criteria

Patients who met any of the following criteria were excluded: (1) lower extremity denervation; (2) lower extremity amputation or congenital limb hypoplasia; (3) severe acute trauma to the lower extremities, including active bleeding or unstable fractures; (4) skin damage, infection, bleeding, or hematoma near the acupuncture site on the lower extremities; (5) the presence of implantable medical devices, such as pacemakers, implantable cardioverter defibrillators (ICDs), or deep brain stimulators (DBSs); (6) pregnancy or breastfeeding; (7) an expected ICU stay of less than 48 h; (8) opting for palliative care instead of active treatment; (9) concurrent participation in another RCT; (10) refusal or inability to provide informed consent; (11) immunosuppressive status, including congenital immunodeficiency, acquired immune deficiency syndrome (AIDS), organ transplantation, long-term immunosuppressant use, or tumor radiotherapy/chemotherapy within 1 month; and (12) ICU physician evaluation indicating an anticipated survival prognosis of less than 7 days, making completion of the acupuncture treatment course unfeasible.

### Randomization and Allocation Concealment

Eligible participants will be randomly assigned to the EA group or the sham-EA group at a ratio of 1:1 through dynamic block randomization. Computer-generated randomization sequences will be produced by an independent investigator and secured in sequentially numbered, opaque, sealed envelopes. Afterward, a nonblinded acupuncturist will access the allocation assignment by opening envelopes in sequential order and subsequently administering the corresponding intervention. To maintain blinding in patients, outcome assessors, and statisticians, the allocation sequence will be concealed until the end of the study.

### Blinding and Masking

In this study, participants and outcome assessors will be blinded to group assignment. To ensure this, we will establish standard EA procedures to ensure that both groups receive identical treatment protocols (e.g., acupuncture time, frequency, and manipulation). Participants will undergo EA treatment and outcome assessments at separate times each day. Acupuncturists will be instructed not to reveal the patients’ group allocation to the outcome assessor and will sign confidentiality agreements. As acupuncturists perform acupuncture by manipulating needles, masking them is not feasible. Given that sepsis patients often have altered mental status, assessment of patient blinding is not applicable to this study.

### Interventions

**Fig. 2:**
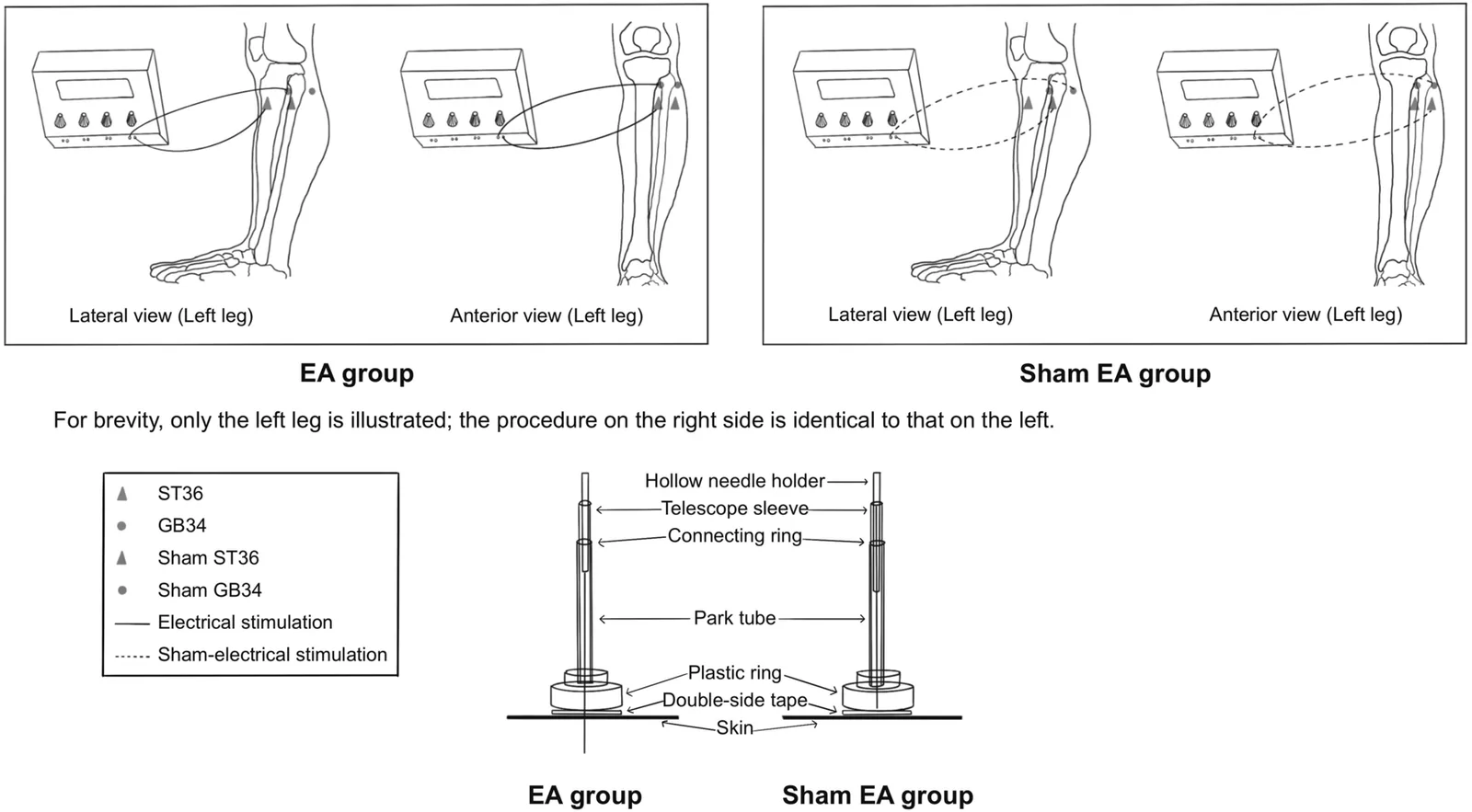
The acupoints and operation methods of the EA group and Sham EA group

The treatment protocol was developed with reference to studies published in *Nature*, *Neuron*, and related clinical research literature, incorporating the opinions of experts in acupuncture and critical care. A detailed list of the experts is provided in supplement 1 (Table S1). EA or sham EA treatment will be performed by qualified acupuncturists, who have a traditional Chinese medicine (TCM) certificate, and who will independently perform clinical treatment for more than two years. Before the treatment begins, acupuncturists should have undergone centralized training to ensure uniformity in the acupuncture procedures. EA conditions and adverse reactions will be recorded after each EA or sham EA on the acupuncture record form. Details of the procedures for the EA group and the sham EA group are presented in Fig. 2.

#### EA Group

Participants in the EA group will receive acupuncture at bilateral acupoints ST36 and GB34. In patients in the supine position, after sterilization, sterile adhesive pads will be placed on the bilateral ST36 and GB34. Afterward, the acupuncturist inserted sharp needles into the skin of acupoints ST36 and GB34 approximately 20 to 30 mm through the adhesive pads, followed by manual manipulation of each needle for 10 s to induce sensation of deqi (a composite of sensations including soreness, numbness, distention, heaviness, and other sensations). Afterward, paired electrodes from the EA apparatus will be attached transversely to the needle handles at bilateral ST36 and GB34. EA stimulation lasted for 30 min, with a continuous wave of 10 Hz and a current intensity of 1 mA (preferably with the skin around the acupoints shivering mildly without pain). Participants will receive EA sessions once daily for 6 consecutive days.

Sterile, disposable Hwato-type acupuncture needles (Suzhou Medical Supplies Factory Co., Ltd., China) 0.30 mm in diameter and 40 mm in length were used. EA stimulation was delivered using a Hwato SDZ‑V EA device.

#### Sham EA group

Participants in the sham EA group will receive sham EA with a noninsertive placebo needle on sham acupoints. The sham ST36/GB34 point is located at 1 cun (approximately 20 mm) lateral to acupoint ST36/GB34. The procedures, electrode placements, and other treatment settings will be the same as those in the EA group but with no skin penetration or electricity output*.*

#### ICU Standard Therapy

All participants received standard ICU therapy, which was developed on the basis of expert consensus [28]. Routine treatment includes source control of infection, antibiotics and/or other anti-infective measures, sedation and analgesia, fluid management, mechanical ventilation, renal replacement therapy, nutritional support and so forth. EA sessions will be scheduled within a flexible daily window, coordinated with bedside staff. Routine ICU care and emergency procedures take priority. If a session cannot be completed because of prone positioning or other interventions, it will be recorded as a protocol deviation.

#### Criteria for Discontinuing the Allocated Intervention

EA treatment can be ended if the following conditions occur: (1) The patient is excluded from the sepsis diagnosis during treatment because of concealment or failure to provide accurate medical information and past medical history by the patient or their family. (2) The patient experience serious adverse events during or after treatment. (3) The patient exhibit poor compliance or refuse to continue treatment.

### Study Endpoints

#### Primary Endpoint

In this study, ICU attending physicians who are blinded to treatment group assignments will be responsible for the outcome measures. The primary outcome of this study is the change in mean total SOFA score on day 7 after randomization compared with baseline (24 h before randomization). The SOFA score is a simple but effective method to describe organ dysfunction/failure in sepsis or other critically ill patients [29]. The scoring criteria for the SOFA score are detailed in supplement 1 (Table S2).

#### Secondary Endpoints

Participants will be followed up until day 28 after randomization. Secondary outcomes include the following: (1) all-cause mortality on day 28; (2) all-cause ICU mortality on day 28; (3) ICU-free days to day 28; (4) hospital-free days to day 28; (5) ventilator-free days to day 28; (6) vasopressor-free days to day 28; (7) continuous renal replacement therapy (CRRT)-free days to day 28; (8) changes in SOFA total scores at days 8 and 9; (9) changes in each item of the SOFA score (i.e., the ratio of arterial oxygen partial pressure to fractional inspired oxygen ratio (PaO_2_/FiO_2_), ventilator use, platelet counts, total bilirubin, mean arterial pressure, vasopressor use, Glasgow Coma Scale score, creatinine level, and urine volume) from baseline to day 9; (10) changes in serum biomarkers and related cellular assays on day 3 and day 7 compared with baseline; and (12) changes in the liquid equilibrium parameters, including daily fluid intake, output and fluid balance from baseline to day 6 after randomization.

### Safety Assessments

In reference to the literature concerning acupuncture-related adverse reactions, outcome assessors will record adverse events (AEs) during and after each treatment. The frequency of AE occurrence will be analyzed and evaluated. In this trial, AEs are defined as follows: (1) Any clinically significant adverse medical event occurring in patients receiving EA or undergoing study procedures that deviates from the expected clinical course of sepsis patients. (2) Any clinically significant adverse medical event considered related to EA or the study procedure, regardless of its alignment with the expected clinical course of sepsis patients. “Response to EA” refers to an adverse event for which a causal relationship with EA cannot be excluded.

In this study, acupuncturists evaluated the safety of acupuncture, and any AEs associated with acupuncture, including bleeding, subcutaneous hemorrhage, serious pain, local infection, and other unexpected adverse reactions, were documented. If AEs occur, the EA or sham EA ends, and experienced ICU doctors and acupuncturists promptly take necessary steps to ensure patient well-being.

### Data Collection and Management

Within 24 h of consent confirmation, the investigators will perform baseline assessments and collect necessary demographic and epidemiological data from the medical records system. Record forms will record information such as age, sex, weight, height, medical history, ICU admission source and classification, infection source, pathogen type, vital signs, baseline SOFA score, and relevant biomarkers.

During the treatment period, all of the aforementioned outcome measures and safety indicators will be recorded. All the reasons for and details of participant discontinuation or withdrawal will also be systematically recorded. All paper-based data will be documented in the case report form (CRF).

Data will be managed through the Tongji Hospital YiDuCloud Follow-Up Platform. Team members have generated electronic case report form (eCRF) on the basis of paper reports and annotations. The data collected from the CRFs will be entered into the system by an investigator.

Upon data submission, the database will be locked. Any postlock modifications must receive principal investigator approval, be signed off by the data entry staff, and be recorded by the system. All records, both paper and electronic, will be retained for at least five years after publication.

### Monitoring and Quality Control

Experts from diverse fields, including acupuncture, intensive care, statistics, and public health, collaboratively developed the trial protocol. If revisions are necessary, an updated protocol will be resubmitted for ethics review.

To ensure study quality, a standardized training program will be provided to all team members prior to recruitment, covering patient screening methods, EA administration, data collection and entry, and an overview of the database. After initiation, regular monitoring is conducted. Quality control for this study will be conducted independently by designated personnel at Wuhan Jinyintan Hospital, a tertiary academic medical center that is not otherwise involved in the research, to evaluate safety parameters and ensure protocol compliance. Comprehensive audits were conducted at least semiannually, with random sampling covering no less than 50% of the total cases. Any data modifications will be traceable via the YiDuCloud Follow-Up Platform. Reasons and details for drop-outs or withdrawals will be documented.

All acupuncture interventions are conducted under continuous ICU monitoring to ensure compliance with the treatment protocol. Additionally, randomized video recordings of more than 30% of treatment sessions will be archived and maintained for two years following study completion to allow for quality control verification.

### Statistical Analysis Plan

#### Sample Size Calculation

The sample size for the study is determined on the basis of the power of the hypothesis test for the primary endpoint (a reduction in the SOFA total score on day 7 postrandomization). Given the limited prior data on the expected effect size, a conventional fixed sample size design would risk insufficient power or inefficient resource use. Therefore, this trial adopts a sequential design, allowing dynamic sample size adjustment based on conditional power re‑estimation at an interim analysis while strictly controlling the overall type I error. The calculation assumes a 1:1 group allocation, an expected mean reduction difference of 1.2 between the EA and sham-EA groups, a common standard deviation of 3.2, a one-sided type I error rate (α) of 2.5%, and 90% power (β = 0.10). On the basis of these parameters, a fixed-sample design requires 300 patients (150 per group).

A single interim analysis will be conducted after 50% of the planned patients have completed the day 7 assessment or discontinued the study. Stopping boundaries are designed to allow early termination for efficacy or futility (nonbinding) and will be determined using a Lan-DeMets spending function approximating O’Brien-Fleming boundaries [30]. Accounting for the group sequential design, the sample size inflation factor is 1.025, resulting in a total sample size of 308 patients (154 per group).

If the study does not stop at the interim analysis, sample size re-estimation will be performed by an unblinded statistician. Conditional power will be calculated on the basis of the observed between-group difference and standard deviation among the first 154 patients. If the conditional power is greater than or equal to 90%, enrollment will proceed to 308 patients. If the conditional power is less than 90%, the sample size may be increased to 500 participants, with a minimum of 308 participants retained. The final sample size will be determined on the basis of statistical considerations, budget, enrollment feasibility, and ethical factors.

#### Statistical Analysis

Statistical analysis will be performed using SAS (version 9.4 or higher) or other validated statistical software (e.g., R). The analysis will be based on predefined populations: the intention-to-treat (ITT) population, which includes all randomized participants who received at least one intervention, and the per protocol (PP) population, which is a subset of the ITT population excluding those with major protocol violations. Primary efficacy analyses will be conducted on both the ITT and PP populations, while baseline demographic data, secondary efficacy analyses, and safety evaluations will be based on the ITT population.

The primary hypothesis will be tested at a one-sided significance level of 0.025, and all other hypotheses will be tested at a two-sided significance level of 0.05. Confidence intervals will be reported at the 95% level. Continuous variables will be summarized using means, standard deviations, medians, and ranges, while categorical variables will be presented as counts and percentages.

The primary endpoint is the reduction in the SOFA score on day 7. The hypothesis is that compared with the sham-EA group, the EA group will have a greater reduction in SOFA score. This hypothesis will be tested using analysis of covariance (ANCOVA) to adjust for baseline SOFA scores.

The study uses an adaptive group sequential design, allowing for early stopping and sample size re-estimation while controlling Type I error inflation. The study is divided into two stages: Stage 1 includes the first 154 randomized subjects for interim analysis, and Stage 2 includes all subsequent subjects. ANCOVA models are applied to each stage separately to estimate Z-statistics, defined as Zᵢ = $\frac{{\widehat{\delta }}_{i}}{se\left({\widehat{\delta }}_{i}\right)}$ (*i* = 1, 2), where ${\widehat{\delta }}_{i}$ is the estimated least squares mean difference and $se\left({\widehat{\delta }}_{i}\right)$ is the standard error.

At the interim analysis, a one-sided *P* value (*P*₁) will be calculated on the basis of Stage 1 data. If *P*₁ is less than 0.0015, the study can stop early for efficacy. If *P*₁ exceeds 0.3947, the study may stop for futility. However, since the futility boundary is nonbinding, enrollment may continue without inflating Type I error. If the study proceeds after the interim analysis, Stage 1 and Stage 2 populations will be analyzed separately to obtain Z₁ and Z₂, and a combined weighted statistic Z = ${w}_{1}{Z}_{1}+{w}_{2}{Z}_{2}$ will be calculated, where ${w}_{1}={w}_{2}=\sqrt{0.5}$ [31]. The final one-sided *P* value derived from Z is 0.0245; if it is less than 0.0245, the null hypothesis is rejected.

In this trial, interim analysis data will be extracted by an independent researcher who is not involved in participant recruitment, randomization, follow‑up, or data entry. Sample size re‑estimation will then be performed by a separate independent unblinded statistician. The results will be reported solely to an independent data monitoring committee (DMC). The study team and clinical staff will remain blinded to the interim results. Unblinded statisticians cannot participate in subsequent protocol amendments, and the DMC may recommend sample size adjustments without disclosing specific results.

For missing data at the primary endpoint, if a patient dies within 7 days, the SOFA score on the day of death will be set to 24 (the highest possible score), and the SOFA score on subsequent days will also be set to 24. Other missing values in the primary endpoint will be imputed using the multiple imputation (MI) method.

### Patient and Public Involvement

Patients will not be involved in the design, conduct, or reporting of this study.

## Discussion

Sepsis is a highly fatal condition that significantly impairs patients’ quality of life. Traditional treatments have limited efficacy, underscoring the need for novel therapeutic strategies to reduce mortality and improve prognosis. Advances in understanding sepsis pathogenesis have emphasized the role of organ function impairment, making the SOFA score a key diagnostic criterion [1, 28, 32]. Moreover, the SOFA score has emerged as a progressively significant instrument for evaluating the efficacy of novel therapeutic agents in exploratory sepsis trials [33]. The unique therapeutic effects of acupuncture have been recognized by multiple countries and international organizations, with millions of patients using it to manage pain and inflammation and to regulate immune homeostasis [34, 35]. However, clinical evidence for the use of EA in the treatment of organ dysfunction among patients with sepsis remains insufficient. Therefore, we are conducting this prospective trial to assess the efficacy and safety of early EA treatment for sepsis by analyzing changes in the SOFA score.

The ST36 acupoint is anatomically located on the anterolateral aspect of the lower leg, specifically 3 cun below Dubi (ST35, the depression lateral to the patellar ligament), belonging to the Stomach Meridian of Foot-Yangming. At deeper tissue levels, this region contains branches of the deep peroneal nerve, which has been experimentally demonstrated to mediate the therapeutic effects of EA in improving sepsis outcomes [21]. In murine models, compared with EA at ST25, low-intensity EA (0.5 mA) at ST36 resulted in greater reductions in inflammation and mortality [25]. A systematic review by the research team further highlighted the importance of ST36 in sepsis treatment [23]. Thus, for this study, ST36 was selected as the primary acupoint, with GB34 as a complementary acupoint on the basis of traditional Chinese medicine. Given that high-intensity EA (3 mA) showed reduced efficacy and greater limitations than low-intensity EA (0.5 mA) in murine studies, this trial set the EA intensity at 0.5–1.0 mA to ensure patient comfort and to achieve slight needle tremors.

In clinical acupuncture research, the blinding methodology and control group design remain persistent challenges. An ideal placebo acupuncture intervention should simultaneously satisfy two criteria: physiological inertness and perceptual equivalence to verum acupuncture. Some authors have suggested that sham acupuncture may result in residual physiological activity, frequently eliciting substantial nonspecific therapeutic responses [36–38]. To observe the specific effects of EA on sepsis, our study will implement a standardized nonpenetrating placebo needle protocol for the sham control group. Both the experimental and control arms will receive identical procedural interventions, following the methodological framework established in our prior migraine research [39].

Owing to the heterogeneity of sepsis, to prevent inaccuracies in sample size estimation, our prospective study adopts an adaptive group sequential trial design. This approach has been applied in sepsis research but represents the first innovative use in studies evaluating EA treatment for sepsis [40, 41]. Treatments targeting a single mediator often demonstrate limited effectiveness in sepsis management [42–46]. In contrast, multitarget therapeutic approaches have yielded more promising results in some clinical trials [47, 48]. EA’s ability to target multiple inflammatory mediators and immune components early in the inflammatory cascade supports its potential to improve sepsis outcomes.

Owing to the nature of acupuncture, acupuncturists cannot be blinded. Therefore, we cannot fully eliminate the potential impact of the acupuncturist’s subjective behavior on the study outcomes. To partially address this issue, acupuncturists will perform the same procedure for both groups, which may help compensate for the lack of blinding. Additionally, the heterogeneity of sepsis means that routine treatments may vary, potentially influencing the results. However, combining acupuncture with real-world clinical treatment allows for a more realistic assessment of its effectiveness. Future studies will explore the immunological mechanisms of EA in the treatment of specific types of sepsis.

## Trial Status

The study began on 11 February 2025 and is anticipated to be completed on 31 August 2026. To date, 136 participants have been enrolled, and 129 have completed all the visits.

## Supplementary Information

Below is the link to the electronic supplementary material.

Supplementary file1 (PDF 145 KB)

## Data Availability

Trial data will be shared on reasonable request post-publication, contingent on completion of a data use agreement.
