# Supplementary material for: Electroacupuncture as Adjunctive Therapy for Organ Dysfunction Among Chinese ICU Patients with Sepsis: Study Protocol for a Randomized Clinical Trial
Source: Curr Med Sci. 2026 Jul 23;46(4):1127–36. doi: 10.1007/s11596-026-00225-5 (PMC13503491; doi:10.1007/s11596-026-00225-5)
Supplement: Supplementary file 1 [file 11596_2026_225_MOESM1_ESM.pdf]

Table S1

Treatment Protocol Development Committee

Name	Title	Specialty
Shusheng Li	Professor	Critical Care Medicine
Shenghao Tu	Professor	Integrated Traditional Chinese and Western Medicine
Yi Bian	Associate Chief Physician	Critical Care Medicine
Lingling Yu	Postdoctoral Research Fellow	Integrated Traditional Chinese and Western Medicine

Table S2

The SOFA score

SOFA score	1	2	3	4
Respiration	< 400	< 300	<200	<100
PaO 2/FiO2, mmHg			-------with respiratory support-----	
Coagulation				
Platelets x $10^3/mm^3$	< 150	< 100	< 50	< 20
Liver				
Bilirubin, mg/dl	1.2-1.9	2.0-5.9	6.0-11.9	>12.0
(μ mol/1)	(20-32)	(33-101)	(102-204)	(< 204)
Cardiovascular			Dopamine > 5	Dopamine > 15
Hypotension		Dopamine ⩽ 5	or epinephrine ⩽ 0.1	or epinephrine > 0.1
	MAP < 70 mmHg	or dobutamine (any dose) [a]	or norepinephrine ⩽ 0.1	or norepinephrine > 0.1
Central nervous system				
Glasgow Coma Score	13-14	10-12	6-9	<6
Renal			3.5-4.9	3.5-4.9
Creatinine, mg/dl	1.2- 1.9	2.0 - 3.4	(300 - 440)	(300 - 440)
(gmol/1) or urine output	(110 - 170)	(171 - 299)	or < 500 ml/day	or < 500 ml/day
